# Protective effects of *Quercus acuta* Thunb. fruit extract against UVB-induced photoaging through ERK/AP-1 signaling modulation in human keratinocytes

**DOI:** 10.1186/s12906-021-03473-1

**Published:** 2022-01-04

**Authors:** Ji-Ae Hong, Donghyuk Bae, Kyo-Nyeo Oh, Dool-Ri Oh, Yujin Kim, Yonguk Kim, So Jeong Im, Eun-jin Choi, Seul-gi Lee, Moonjong Kim, Changsik Jeong, Chul Yung Choi

**Affiliations:** 1 Jeonnam Bioindustry Foundation, Jeonnam Institute of Natural Resources Research, Jeollanamdo, South Korea; 2 School of Biological Sciences and Biotechnology, College of Natural Sciences, Chonnam National University, 77 Yongbong-ro, Buk-gu, Gwangju, 61186 Republic of Korea; 3 Department of Biomedical Science, College of Natural Science, Chosun University, 309, pilmun-daero, Dong-gu, Gwangju, 61452 Republic of Korea

**Keywords:** *Quercus acuta* Thunb. fruit, Mitogen-activated protein kinase, Extracellular signal-regulated kinases, Activator protein 1, Matrix metalloproteinase-1, Ultraviolet B, Photoaging

## Abstract

**Background:**

*Quercus acuta* Thunb. (Fagaceae) *or* Japanese evergreen oak is cultivated as an ornamental plant in South Korea, China, Japan, and Taiwan and used in traditional medicine. The acorn or fruit of *Quercus acuta* Thunb. (QAF) is the main ingredient of acorn jelly, a traditional food in Korea. Its leaf was recently shown to have potent xanthine oxidase inhibitory and anti-hyperuricemic activities; however, there have been no studies on the biological activity of QAF extracts. Solar ultraviolet light triggers photoaging of the skin, which increases the production of reactive oxygen species (ROS) and expression of matrix metalloproteinase (MMPs), and destroys collagen fibers, consequently inducing wrinkle formation. The aim of this study was to investigate the effect of water extracts of QAF against UVB-induced skin photoaging and to elucidate the underlying molecular mechanisms in human keratinocytes (HaCaT).

**Methods:**

In this study, we used HPLC to identify the major active components of QAF water extracts. Anti-photoaging effects of QAF extracts were evaluated by analyzing ROS procollagen type I in UVB-irradiated HaCaT keratinocytes. Antiradical activity was determined using 2,2-diphenyl-1-picrylhydrazyl and 2,20-azino-bis (3-ethylbenzothiazoline-6-sulphonic acid) assays. The expression of MMP-1 was tested by western blotting and ELISA kits. QAF effects on phosphorylation of the MAPK (p38, JNK, and ERK) pathway and transcription factor AP-1, which enhances the expression of MMPs, were analyzed by western blots.

**Results:**

We identified two major active components in QAF water extracts, gallotannic acid and ellagic acid. The QAF aqueous extracts recovered UVB-induced cell toxicity and reduced oxidative stress by inhibiting intracellular ROS generation in HaCaT cells. QAF rescued UVB-induced collagen degradation by suppressing MMP-1 expression. The anti-photoaging activities of QAF were associated with the inhibition of UVB-induced phosphorylation of extracellular signal-regulated kinase (ERK) and activator protein 1 (AP-1). Our findings indicated that QAF prevents UVB-induced skin damage due to collagen degradation and MMP-1 activation via inactivation of the ERK/AP-1 signaling pathway. Overall, this study strongly suggests that QAF exerts anti-skin-aging effects and is a potential natural biomaterial that inhibits UVB-induced photoaging.

**Conclusion:**

These results show that QAF water extract effectively prevents skin photoaging by enhancing collagen deposition and inhibiting MMP-1 via the ERK/AP-1 signaling pathway.

**Supplementary Information:**

The online version contains supplementary material available at 10.1186/s12906-021-03473-1.

## Background

Skin aging is characterized by the loss of structure, wrinkles, pigmentation, drying, degradation of collagen, and physiological functions, and occurs due to two biochemical processes: intrinsic and extrinsic (photoaging) aging [1–3]. Intrinsic aging is an inevitable process that occurs over time and is highly correlated with genetic factors [4, 5]. In contrast, extrinsic aging is caused by several hazardous environmental factors. Therefore, the skin serves as the first line of defense protecting the body against extrinsic factors and is continuously in contact with toxic chemicals, infectious agents, and ultraviolet radiation [6]. Repeated exposure to solar ultraviolet (UV) light, particularly UVB, is the primary cause of extrinsic skin aging and induces changes in the epidermis [7]. Photodamaged skin is characterized by dappled discoloration, thickened epidermis, brown spots, loss of elasticity, deep wrinkles, and retarded skin cell growth associated with slower wound healing [8–11]. UVB causes sunburn, inflammatory response, and immune suppression, as well as overproduction of reactive oxygen species (ROS) in the skin.

UVB-induced intracellular ROS activates mitogen-activated protein kinase (MAPK) signaling pathways through the phosphorylation of extracellular signal-regulated kinase (ERK), p38, and c-Jun N-terminal kinase (JNK) [12] important role in signaling pathways, and ultimately participate in the induction of matrix metalloproteinase (MMP) activation [9, 13–15]. In dermal fibroblasts, ERK phosphorylation mediates the inhibition of type I collagen synthesis [16, 17]. Type I collagen, the primary component of the extracellular matrix (ECM) in the skin, is synthesized and secreted by dermal fibroblasts [18]. MAPK regulates the activation of the activator protein (AP)-1 signaling pathway, which is a major regulatory protein consisting of two subunits, c-Fos and c-Jun, and is strongly implicated in mediating the photoaging response [19–22]. MMPs are a family of structurally related zinc-dependent endopeptidases that can degrade all components of ECM proteins and connective tissues [23, 24]. Notably, in dermal fibroblasts, the activation of MMP-1 (known as collagenase) causes collagen fragmentation and functional alterations [18, 25]. In addition, the degraded collagen fragments produced by MMP-1 downregulate new collagen synthesis in vitro and in vivo. [26] Thus, regulation of ROS formation and the signaling cascade related to UV irradiation could have a profound impact on the treatment of skin photoaging [27]. ROS scavengers play a role in photoaging activities and are possibly mediated by the attenuation of MMP production [24] Plants and their components have ROS scavenging activity.

Quercus acuta is cultivated as an ornamental and dietary plant in South Korea, China, Japan, and Taiwan [28]. Quercus acuta Thunb. fruit (QAF) (acorn), a traditional food in Korea, is the main ingredient of acorn jelly [29]. This plant has long been used as a traditional medicine with beneficial effects for the treatment of various diseases [28]. Previous studies have demonstrated that the ethyl acetate extract of QA leaf has potent xanthine oxidase inhibitory and anti-hyperuricemic activities [28, 30]. To the best of our knowledge, only a few studies have investigated the pharmacological activity of various QA extracts and there have been no studies on the biological activity of QAF extracts. Recently, dietary and nutritional factors have gained increasing scientific attention for their potent protective effects against UVB-induced skin damage [31]. Therefore, in this study, we elucidated the mechanism by which QAF prevents UVB-induced damage to keratinocytes.

## Methods

### Chemicals and reagents

Standard gallotannic acid (100% purity) was purchased from the United States Pharmacopeia (Twinbrook Parkway, Rockville, MD, USA). The ellagic acid standard (95% purity), formic acid, 3-(4,5-dimethylthiazol-2-yl)-2,5-diphenyltetrazolium bromide (MTT), 20,70-dichlorodihydrofluorescein diacetate, DPPH, hydrogen peroxide (H_2_O_2_), epidermal growth factor (EGF) and β-actin antibodies were purchased from Sigma-Aldrich (St. Louis, MO, USA). Methanol and water were supplied by JT Baker (Deventer, Holland). Antibodies against ERK, phospho-ERK, p38, phospho-p38, JNK, phospho-JNK, c-Jun, phospho-c-Jun, c-fos, and phospho-c-fos were purchased from Cell Signaling Technology (Beverly, MA, USA), and antibodies against MMP-1 were purchased from Abcam (Cambridge, UK). Horseradish peroxidase-labeled secondary antibodies were obtained from Santa Cruz Biotechnology (Santa Cruz, CA, USA). Polyvinylidene fluoride membrane (Immobilon-P) was obtained from Millipore Co. (Billerica, MA, USA). Human MMP-1 ELISA kit was purchased from R&D Systems (Minneapolis, MN, USA). Procollagen type I (cat. #. MK101) kit was purchased from TaKaRa Bio Inc. (Shiga, Japan). Enhanced chemiluminescence reagent, protein assay, tween-20, acrylamide, ammonium persulfate, and skim milk were purchased from Bio-Rad Laboratories (Hercules, CA, USA). Dulbecco’s modified Eagle medium (DMEM), fetal bovine serum, phosphate-buffered saline (PBS), penicillin, and streptomycin were purchased from Gibco (Carlsbad, CA, USA). All other chemicals and reagents were of guaranteed analytical grades.

### Preparation of plant extract

QAF were collected from Jeollanamdo Wando arboretum, Wando, Korea, and all activities were permitted for the collection of plant material. All locations of plant collection were Wando-arboretum-owned and the field studies did not involve endangered or protected species. The collection of the plant material complied with local regulations. The voucher specimens of the plant(JINR1008) and extracts (JINR2008) were deposited in the Laboratory of Jeonnam Institute of Natural Resources Research (JINR), Jeollanamdo, South Korea. Dried QAF (1,000 g) was placed in a bottle, 20 L of distilled water was added, and extraction was performed at 100 °C for 4 h using a reflux extractor. Then, the extract was filtered (Whatman No. 41); the filtrate was evaporated using a rotary evaporator and freeze-dried at -50 °C for 48 h using a freeze dryer. A total of 54 g (5.4%) of water extract powder was obtained using the above method and the extract was stored at 4 °C until further use. The sample used in the present study was also used in a clinical trial on human skin wrinkle improvement effect (clinical trial registration number TEK-2020-000581), which was approved by the Korea Testing and Research Institute (KTR) (KTR IRB approved number: TEK-KUME1WB-2020).

### Analysis of QAF extract

The QAF extract analysis was performed using the Waters series high-performance liquid chromatography (HPLC) system (e2695, Waters Corporation 34 Maple Street Milford, MA, USA) equipped with a photodiode array detector (2998) and Triart-C18 column (250 mm × 4.6 mm, 5 μm, YMC, Japan). The detection wavelength was set at 254 nm for the water extract, while the column thermostat was maintained at 35 °C. Mobile phase A was methanol and mobile phase B was water (containing 0.1% formic acid) with the following elution profile: initial, 15% A; 5–10 min, 15%–30% A; 10–17 min, 30%–40% A; 17–22 min, 40%–50% A; 22–35 min, 50% A; 35–43 min, 50%–100% A; 43–47 min, 100% A; 47–50 min, 100%–15% A; and 50–55 min, 15% A. The flow rate was 1 mL/min, and the injection volume was 10 μL.

### Cell culture

The HaCaT cell line was obtained from Prof. Chulyuog Choi at Chosun University, Korea. HaCaT keratinocytes cells were cultured in DMEM supplemented with 10% fetal bovine serum, penicillin (50 U/mL), and streptomycin (50 μg/mL). The cells were maintained at 37 °C in an incubator with a humid atmosphere of 5% CO_2_ and 95% air. For UVB irradiation, the cells were exposed to UVB light at a dose of 30 mJ/cm^2^. A UVM-225D Mineralight UV Display Lamp (UVP, Inc.) was used as the UVB source, which emitted light at a wavelength of 302 nm. The strength of UVB radiation was measured using an HD2102-2 UV meter (Delta OHM Srl, Padova, Italy).

### Determination of cell viability

HaCaT keratinocytes cell viability was measured using the MTT assay. HaCaT keratinocytes cells were seeded into 96-well plates (0.5 × 10^5^ cells/well in 100 μL medium) for 24 h and treated with QAF concentration gradient ( 5, 10, 20, or 50 μg/mL) for 24 h. At the end of the sample pre-treatment, the medium was replaced with fresh medium containing MTT (0.5 mg/mL), and incubated for 4 h at 37 °C. Following incubation, the supernatants were removed, dimethyl sulfoxide(DMSO) was added to each well to dissolve the formazan crystals. The OD was determined at 540 nm using a microplate reader (Molecular Devices, Sunnyvale, CA, USA).

### ELISA analysis

MMP-1 activity was examined using an ELISA kit (R&D systems, Inc., Minneapolis, MN, USA). In brief, cells grown to approximately 70 ~ 80% confluence in 48-well culture plates were treated with the indicated concentrations of QAF in serum-free DMEM for 24 h. After incubation, cells were exposed to UVB irradiation (30 mJ/cm^2^). After irradiation, culture media were collected and MMP-1 secretion was analyzed by ELISA kits. Assay was performed according to the manufacturer’s instructions.

### Radical scavenging activity assay

2,2-Diphenyl-1-picrylhydrazyl (DPPH) radical scavenging assays and 2,20-azino-bis(3-ethylbenzothiazoline-6-sulphonic acid) (ABTS) radical scavenging assays were performed to evaluate the hydrogen- and electron-donating capacity of QAF and confirm the antioxidant activity of QAF using previously described methods [32].

### Measurement of intracellular ROS.

An intracellular ROS was measured according to [33]. Briefly, cells were seeded into 96-well plates (0.5 × 10^5^ cells/well) and cultured for 24 h. After overnight incubation, the cells were treated with the indicated concentrations of QAF in serum-free DMEM for 24 h. After incubation, cells were washed with PBS and exposed to UVB irradiation (30 mJ/cm^2)^. After irradiation, cells were incubated for 30 min. After removing the supernatant, 20 μM of DCF-DA was added. After incubation for an additional 30 min, cells were washed 3 times. Fluorescence was measured on a fluorescence plate reader (SpectraMax Gemini EM, Molecular Devices, Sunnyvale, CV, USA).

### Measurement of procollagen type I

Procollagen type I production was measured according to [34]. Procollagen type I (cat # MK101) was produced in HaCaTcell culture media and quantified using ELISA kits according to the manufacturer’s instructions (TaKaRa Bio Inc., Shiga, Japan).

### Western blotting

HaCaT keratinocytes cells were seeded in a 6-well plate at a density of 2 × 10^5^ cells/well for 24 h. The cells were pre-treated with the indicated concentrations of QAF in serum-free DMEM for 24 h. Supernatants were removed, cells were resuspended in lysis buffer (250 mM NaCl,, 25 mM Tris–HCl, 5 mM ethylenediaminetetraacetic acid, 1 mM phenylmethylsulfonyl fluoride, 1% NP-40, 5 mM dithiothreitol, 10 mM NaF, 0.1 mM Na_3_VO_4_, leupeptin, and protease inhibitor). Equal amounts of total proteins were separated using 10% sodium dodecyl sulfate–polyacrylamide gel electrophoresis and transferred to polyvinylidene fluoride (PVDF) membranes. The membranes were blocked with 5% non-fat milk in PBS containing 0.4% Tween-20 (PBS-T) for 1 h at room temperature (20–25 °C). After blocking, membranes were subsequently incubated with the following primary antibodies: ERK, p-ERK, JNK, p-JNK, p38, p-p38, c-Jun, p–c-Jun, c-Fos, p–c-Fos, MMP-1 or β-actin at 4 °C overnight. The membranes were subsequently washed thrice and incubated with diluted horseradish peroxidase-conjugated anti-rabbit IgG secondary antibodies (1:3,000) for 1 h at room temperature (20–25 °C). Detection was performed using a chemiluminescence detection kit (Merck Millipore, Darmstadt, Germany) in accordance with the manufacturer’s instructions. β-actin (Sigma-Aldrich) was used as a loading control for all experiments.

### Statistical analysis

Data are presented as mean ± standard deviation. The results were analyzed using Student’s t-test or one-way analysis of variance using GraphPad Prism 5.0 (GraphPad Inc., San Diego, CA, USA) software program. Statistical significance was set at ^∗^*p* < 0.05, ^∗∗^*p* < 0.01, and ^∗∗∗^*p* < 0.001.

## Results

### Identification of active compounds in QAF extract

**Fig. 1:**
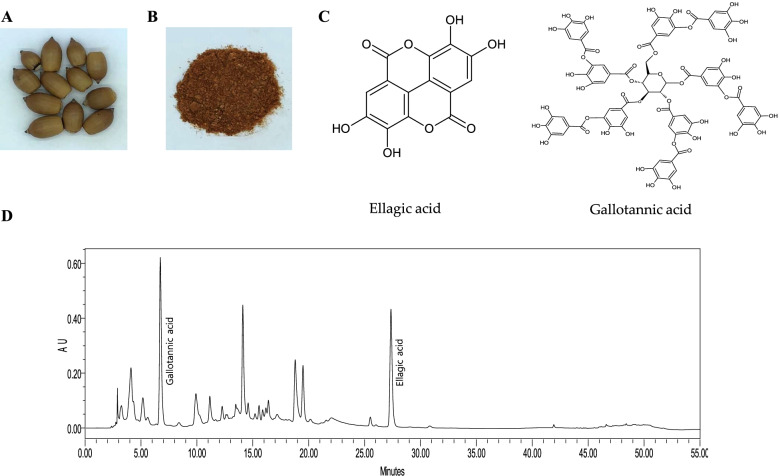
Classic features of *Quercus acuta* Thunb. fruit (QAF) and representative high-performance liquid chromatography (HPLC) chromatogram of a water extract obtained from QAF. (A) Image of QAF, (B) Powder form of QAF aqueous extract, (C) Structural formula of gallotannic acid and ellagic acid, (D) Mobile phase A was methanol and mobile phase B was water (containing 0.1% formic acid) with the following elution profile: initial, 15% A; 5–10 min, 15%–30% A; 10–17 min, 30%–40% A; 17–22 min, 40%–50% A; 22–35 min, 50% A; 35–43 min, 50%–100% A; 43–47 min 100% A; 47–50 min, 100%–15% A; and 50–55 min, 15% A. The flow rate was 1 mL/min, the injection volume was 10 μL, and the detection wavelength was 254 nm. Gallotannic acid and ellagic acid were detected at approximately 6.7 min and 27.3 min, respectively

Classic features of Quercus acuta Thunb. fruit (QAF), powder form of QAF aqueous extract are shown. (Fig. 1A, B). HPLC analysis of QAF aqueous extract showed several peaks. We identified gallotannic acid and ellagic acid as two major constituents of QAF, which were compared with the peaks in the standard graph. The retention times were consistent with standard compounds. This result was consistent with the retention time and absorbance. The retention times of gallotannic acid and ellagic acid in QAF were detected at approximately 6.7 min and 27.3 min, respectively (Fig. 1C, D).

### QAF attenuates UVB-reduced cell proliferation and degradation of collagen in HaCaT keratinocytes

**Fig. 2:**
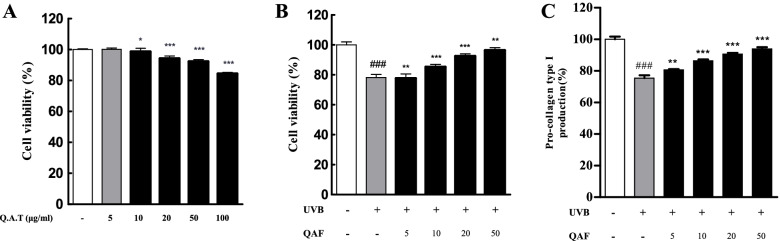
Effects of *Quercus acuta* Thunb. fruit (QAF) on cell proliferation, ultraviolet B (UVB)-reduced cell proliferation, and procollagen type I production in UVB-induced HaCaT keratinocytes. (A) HaCaT cells treated with a range of concentrations of QAF (5, 10, 20, 50, and 100 μg/mL) for 24 h. Cell viability was estimated using MTT assay by measuring the absorbance at 450 nm. (B) Cells were exposed to UVB irradiation (30 mJ/cm^2^) and treated with a range of concentrations of QAF (5, 10, 20, and 50 μg/mL) for 24 h. (C) Cell culture media were collected to determine the levels of procollagen type I. Data are expressed as the mean ± SD. #*p* < 0.05, ##*p* < 0.01, ###*p* < 0.001 versus control group; *p < 0.05, **p < 0.01, ***p < 0.001 versus UVB-treated group

We performed the MTT assay to examine the cytotoxic effects of UVB and QAF on HaCaT cells. QAF treatment did not cause marked cytotoxicity for 24 h (Fig. 2A). Thus, for further cell-based experiments, QAF was used at concentrations of 2.5–50 μg/mL. As shown in Fig. 2B, UVB exposure (30 mJ/cm^2^) reduced viability of HaCaT cells by 80.59% ± 0.97% compared to that of control cells. QAF alleviated cell proliferation (80.88% ± 0.90% at 5 μg/mL, 86.05% ± 1.32% at 10 μg/mL, 92.39% ± 0.66% at 20 μg/mL, and 96.31% ± 1.298% at 50 μg/mL), and protected the cells from the toxic effects of UVB irradiation. Cells exposed to UVB exhibited markedly reduced procollagen type I production at 75.46% ± 1.79%. Production of procollagen type I was significantly improved with QAF treatment (80.62% ± 0.57% at 5 μg/mL, 86.48% ± 0.72% at 10 μg/mL, 90.69% ± 0.78% at 20 μg/mL, and 93.95% ± 1.07% at 50 μg/mL) (Fig. 2C).

### DPPH, ABTS, and ROS scavenging activities

**Fig. 3:**
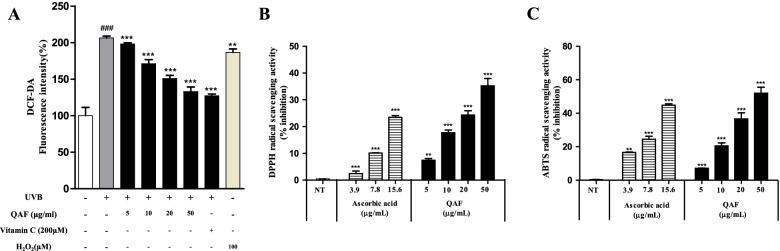
Free radical scavenging activity analysis and intracellular reactive oxygen species (ROS) production in *Quercus acuta* Thunb. fruit (QAF)-treated HaCaT cells. (A) Effects of QAF on ROS production following ultraviolet B (UVB) irradiation. HaCaT cells were pretreated with a range of concentrations of QAF (5, 10, 20, and 50 μg/mL) or vitamin C (ascorbic acid) at 200 μM for 24 h, H_2_O_2_ (100 μM) for 2 h, followed by exposure to 30 mJ/cm2 UVB. After incubation, cells were stained with DCFH-DA (20 μM) for 30 min. Fluorescence was then measured using a fluorescence spectrophotometer. (B) 2,2-Diphenyl-1-picrylhydrazyl (DPPH) and (C) 2,20-azino-bis (3-ethylbenzothiazoline-6-sulphonic acid) (ABTS) radical scavenging activities. DPPH was examined with different concentrations of QAF, and ascorbic acid was used as a standard. Data are expressed as the mean ± SD. #*p* < 0.05, ##*p* < 0.01, ###*p* < 0.001 versus control group; *p < 0.05, **p < 0.01, ***p < 0.001 versus UVB-treated group

UVB exposure increased intracellular ROS generation in HaCaT cells (Fig. 3A, bar 2), which was remarkably suppressed by QAF treatment (Fig. 3A, bars 4–6). Various cell-free antioxidant assay systems, such as DPPH and ABTS radical scavenging assays, were performed to determine the antioxidant ability of QAF. QAF markedly exhibited DPPH and ABTS radical scavenging activities (Figs. 3B and C).

### QAF reduces the protein level and production of MMP-1 in UVB-induced HaCaT keratinocytes

**Fig. 4:**
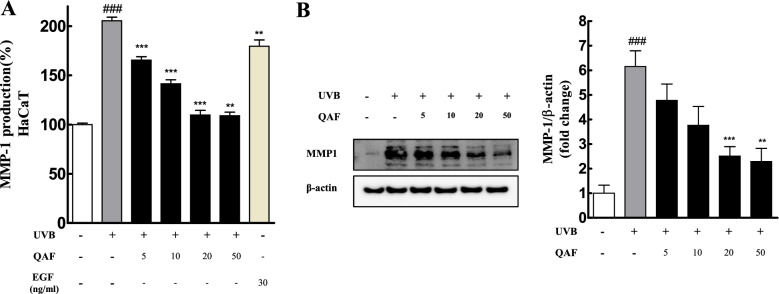
Effects of QAF on matrix metalloproteinases (MMP)-1 expression in UVB-stimulated HaCaT cells. The cells were pretreated with *Quercus acuta* Thunb. fruit (QAF) for 24 h, followed by UVB-irradiation. (A) Cells were seeded and pretreated with a range of concentrations of QAF (5, 10, 20, and 50 µg/mL) and 30 ng/ml EGF, followed by UVB irradiation and cultured for an additional 24 h. The level of MMP-1 released in the cell culture medium was measured using ELISA. (B) Protein expression of MMP-1 was analyzed using western blotting and band intensities were quantified. Cells were seeded and pretreated with a range of concentrations of QAF (5, 10, 20, and 50 μg/mL), followed by UVB irradiation and cultured for an additional 24 h. Data are expressed as the mean ± SD. #*p* < 0.05, ##*p* < 0.01, ###*p* < 0.001 versus control group; *p < 0.05, **p < 0.01, ***p < 0.001 versus UVB-treated group

MMPs are the major endopeptidases that induce collagen degradation, and thus overexpression of MMPs is a major characteristic of photodamaged skin. It is known that UVB light stimulates the protein expression and activity of MMP-1 and its overexpression initiates degradation of collagen types I and III. Therefore, MMP-1 plays an important role in the physiological mechanisms of photoaging [35, 36], and the development of MMP-1 inhibitors is important for anti-aging research. We found that MMP-1 production was increased by UVB irradiation, and QAF significantly inhibited these upregulations in HaCaT keratinocyte cell lines, as measured using enzyme-linked immunosorbent assay (ELISA) (Fig. 4A). The expression level of MMP-1 was elevated in the UVB-irradiated group compared to that in the control; however, QAF effectively attenuated this increase (Fig. 4B). Signal intensities of MMP-1 were quantified using Image Lab software version 6.1 (Bio-Rad).

### QAF suppresses UVB-induced activation of ERK/AP-1 signaling pathways in HaCaT keratinocytes

The molecular mechanisms of photoaging involve complex signaling cascades that are generally initiated by UVB irradiation, which subsequently stimulates the phosphorylation of protein kinase through the MAPK pathway. Phosphorylation of the MAPK (p38, JNK, and ERK) pathway directly activates the transcription factor AP-1, which can enhance the expression of MMPs [37, 38]. AP-1 is a major regulatory protein consisting of two subunits, c-Fos and c-Jun, and is strongly implicated in mediating the photoaging response [19]. Previous studies have described that activation of the AP-1 signaling pathway is regulated by MAPK [20]. Thus, MAPK pathways play an important role in regulating MMP expression [14, 15], and we investigated the pathway through which QAF exhibits its anti-photoaging effects.

**Fig. 5:**
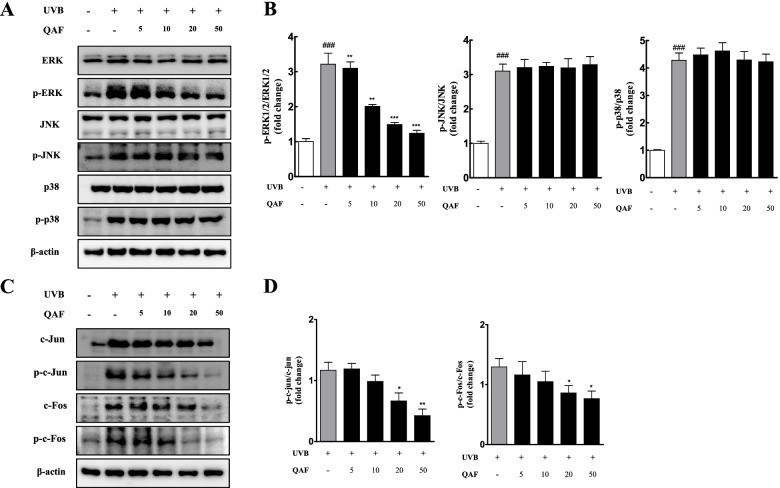
Effects of *Quercus acuta* Thunb. fruit (QAF) on activator protein 1 (AP-1) and mitogen-activated protein kinase (MAPK) signaling pathways in UVB-induced HaCaT keratinocytes. (A) The effects of QAF on the phosphorylation of MAPK activated by UVB. Cells were seeded and treated with a range of concentrations of QAF (5, 10, 20, and 50 μg/mL) for 24 h. Cells were irradiated with UVB at a dose of 30 mJ/cm^2^ and harvested 30 min later. Protein expression was evaluated using western blotting, and (B) the band intensities were quantified. (C) The effects of QAF on the phosphorylation of AP-1 activated by UVB. Cells were treated with a range of concentrations of QAF (5, 10, 20, and 50 μg/mL) for 24 h, irradiated with UVB at a dose of 30 mJ/cm^2^, and harvested 10 h later. Protein expression was evaluated using western blotting, and (D) the band intensities were quantified. Data are expressed as the mean ± SD. #*p* < 0.05, ##*p* < 0.01, ###*p* < 0.001 versus control group; *p < 0.05, **p < 0.01, ***p < 0.001 versus UVB-treated group

To elucidate whether QAF attenuates UV-induced MMP 1 expression in HaCaT cells by influencing the MAPK signaling pathways, the cells were treated with the indicated concentrations of QAF for 24 h before UV irradiation. UVB irradiation leads to the activation of ERK, p38, and JNK. As shown in Fig. 5A, levels of p-p38, p-ERK1/2, and p-JNK were significantly increased by UVB exposure. Treatment with QAF inhibited the levels of p-ERK1/2; however, p-p38 and p-JNK were unaffected. Signal intensities of MAPK were quantified (Fig. 5B) using Image Lab software version 6.1 (Bio-Rad).

**Fig. 6:**
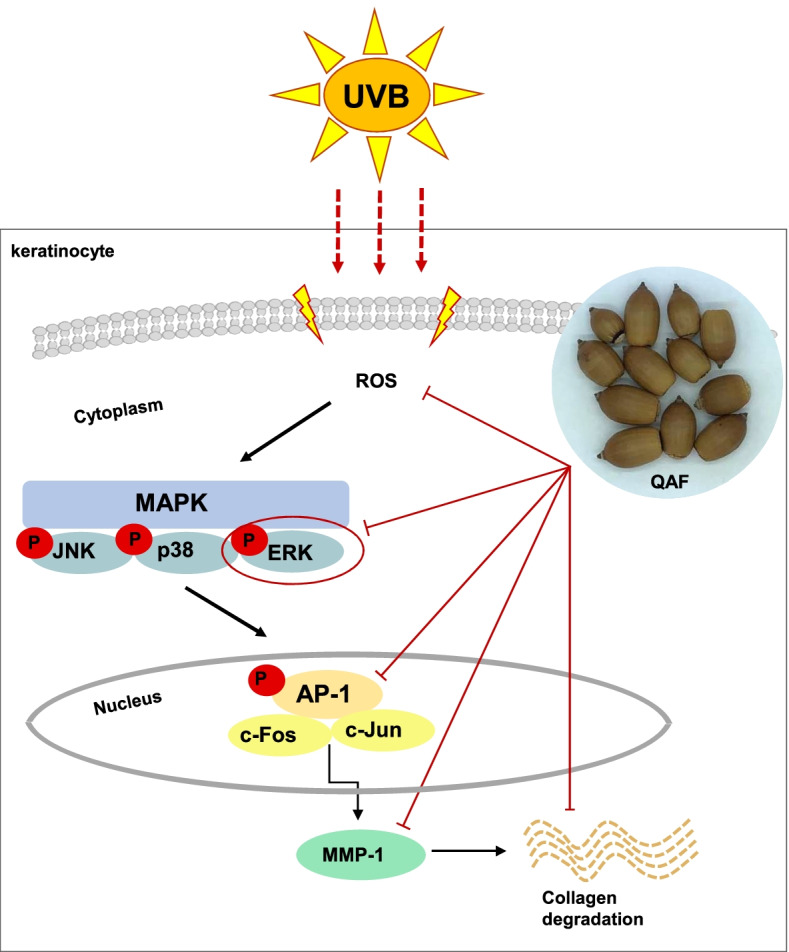
A proposed mechanism of action of *Quercus acuta* Thunb. fruit on ultraviolet B-induced photoaging in HaCaT cells

Previous studies have described that activation of the AP-1 signaling pathway is regulated by MAPK [20–22]. QAF inhibits the phosphorylation of ERK, whose downstream targets are c-Jun and c-Fos [39]. Therefore, to validate whether QAF downregulates AP-1, we treated HaCaT cells with the indicated concentrations of QAF for 24 h before UVB irradiation. UVB exposure enhanced c-Jun and c-Fos expression and phosphorylation. QAF successfully inhibited the protein levels of c-Fos and c-Jun, as well as its phosphorylation (Fig. 5C), and the band intensities were quantified (Fig. 5D). The possible mechanism of action of QAF against photoaging is summarized in Fig. 6.

## Discussion

With an increase in life expectancy, the physical and functional effects of skin aging are increasing. Numerous factors affect skin aging, including UV light and stress [40] most UVB-irradiated skin show cytotoxicity by increasing ROS production, which triggers the skin damage process, impairment of collagen fibers, and an inordinate deposition of abnormal elastin complex [27]. UVB radiation also causes oxidative stress, ROS-mediated DNA damage, and modulation of ECM components, such as MMPs and collagen, thereby expediting skin photoaging [41, 42].

Studies have shown that certain natural topical agents can prevent skin aging [31] recent studies have revealed that certain dietary factors can attenuate skin aging [43, 44]. Therefore, the cosmeceutical market and pharmaceutical industries have focused on numerous herbal extracts with potential anti-inflammatory and antioxidant properties to develop efficacious anti-aging products against photoinduced dermatological diseases [45–47]. Thus, we attempted to identify natural candidates that can inhibit photoaging. Screening studies have shown that QAF is a promising candidate. Koreans widely consume the *Quercus acuta* fruit; however, there have been no studies on the biological activity of QAF.

Our study is the first to determine the anti-photoaging activity of QAF and elucidate the associated molecular mechanisms.

In this study, we found that QAF rescued UVB-induced cytotoxicity and substantially inhibited cellular ROS production in human keratinocytes. Together with procollagen type I, collagen and elastin, the major protein components of the ECM, are responsible for skin elasticity and skin moisture [44]. QAF showed a strong antioxidant activity, and exhibited strong ABTS and DPPH radical scavenging potentials as compared to that of ascorbic acid.

In the skin photoaging process, UVB-induced ROS production upregulates the expression of MMPs, which leads to the degradation of collagen and other ECM proteins [36]. QAF significantly decreased MMP-1 protein secretion in UVB-irradiated HaCaT cells, and acted as an MMP-1 inhibitor.

MAPK pathways play an important role in regulating MMP expression [14, 15] and in this study, we investigated whether QAF influences MMPs via the MAPK signaling pathway. The results indicated that QAF decreased ERK phosphorylation in a dose-dependent manner and attenuated the protein levels of c-Fos and c-Jun, as well as those of their phosphorylated counterparts. Here, the ameliorative effect of QAF on MMP-1 overexpression was likely regulated by the inhibition of ERK/AP-1 signaling activation. Therefore, the effect of QAF on the MAPK signaling pathway may be one of the key mechanisms that suppress the upregulated expression of MMPs caused by UVB irradiation.

Based on these results, it was concluded that QAF may be a potential therapeutic agent to treat skin disorders, such as photoaging, with a promising cosmeceutical and pharmaceutical value.

## Conclusions

Our study showed that QAF effectively prevents skin photoaging by enhancing collagen deposition and inhibiting MMP-1 via the ERK/AP-1 signaling pathway. These results indicate that QAF is a potential therapeutic candidate for improving photoaged skin.

## Supplementary Information

Additional file 1

## Data Availability

The data used and/or investigated during the present study are available from the corresponding author upon reasonable request.

## Abbreviations

ABTS: 2,20-Azino-bis (3-ethylbenzothiazoline-6-sulphonic acid)
AP: Activator protein
DCF: 2,7-Dichlorodihydrofluorescein
DMEM: Dulbecco’s modified Eagle medium
DPPH: 2,2-Diphenyl-1-picrylhydrazyl
ECM: Extracellular matrix
ERK: Extracellular signal-regulated kinase
HPLC: High-performance liquid chromatography
JNK: C-Jun N-terminal kinase
MAPK: Mitogen-activated protein kinase
MMP: Matrix metalloproteinase
MTT: 3-(4,5-Dimethylthiazol-2-yl)-2,5-diphenyltetrazolium bromide
PBS: Phosphate-buffered saline
PBS-T: PBS containing Tween-20
QAF: *Quercus acuta* Thunb.
ROS: Reactive oxygen species
