# Supplementary material for: Protective effects of Quercus acuta Thunb. fruit extract against UVB-induced photoaging through ERK/AP-1 signaling modulation in human keratinocytes
Source: BMC Complement Med Ther. 2022 Jan 4;22:6. doi: 10.1186/s12906-021-03473-1 (PMC8728912; doi:10.1186/s12906-021-03473-1)
Supplement: Supplementary file 1 — Additional file 1 [file 12906_2021_3473_MOESM1_ESM.pdf]

Figure 4B

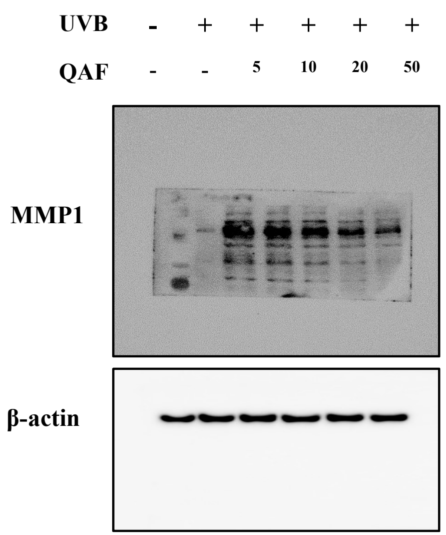

Figure 5A

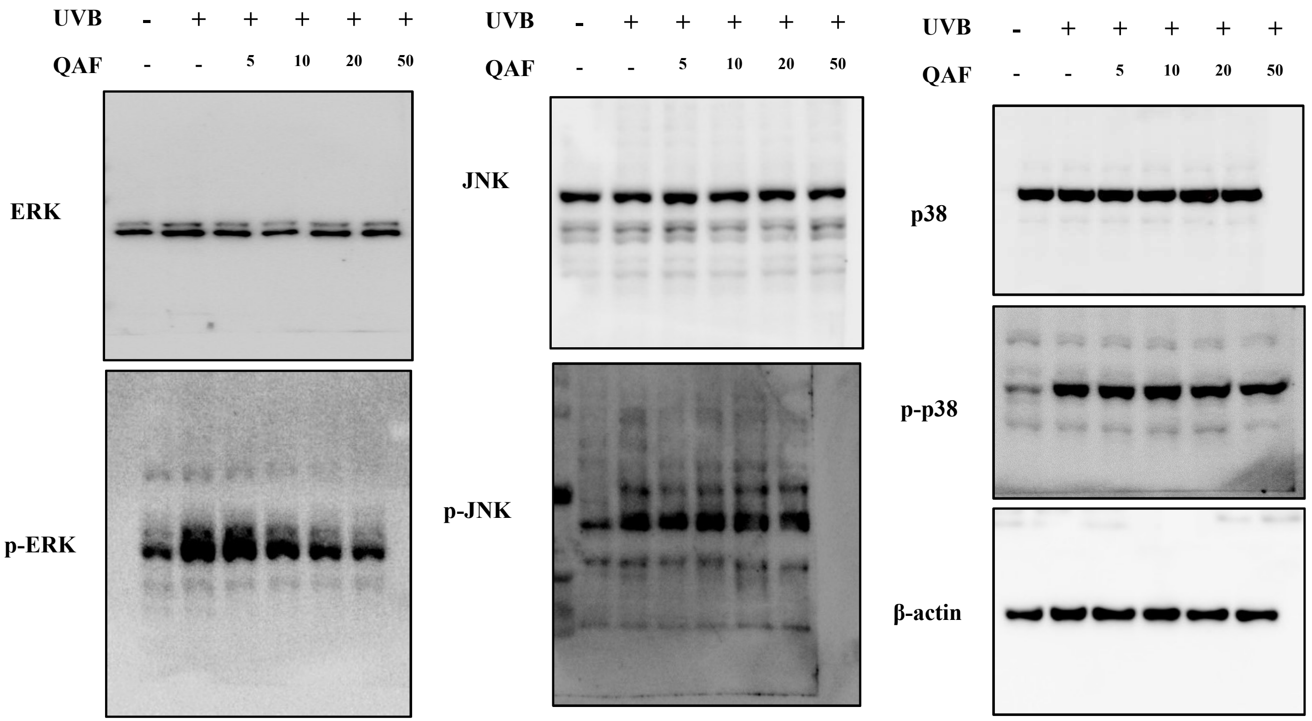

Figure 5C

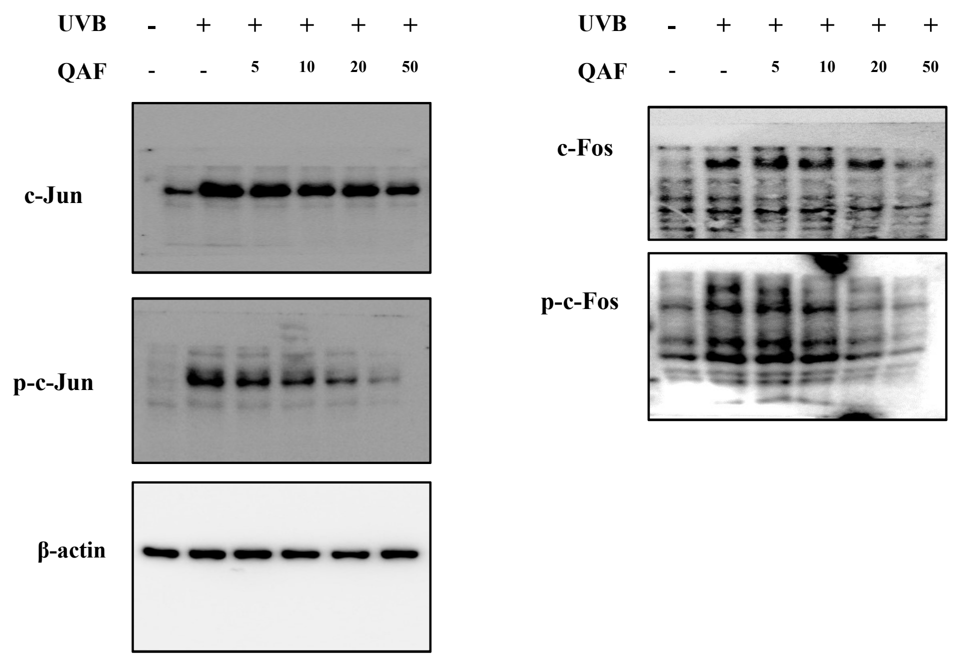
UVB - + + + + +
QAF - - 5 10 20 50
c-Jun
p-c-Jun
β-actin
UVB - + + + + +
QAF - - 5 10 20 50
c-Fos
p-c-Fos
